# Automated external defibrillator: Rhythm analysis and defibrillation on paediatric out-of-hospital cardiac arrest^☆^

**DOI:** 10.1016/j.resplu.2025.100873

**Published:** 2025-01-16

**Authors:** Emma Menant, Delphine Lavignasse, Sarah Ménétré, Jean-Philippe Didon, Xavier Jouven

**Affiliations:** a Université Paris Cité, INSERM U970, Paris Cardiovascular Research Centre (PARCC), Integrative Epidemiology of Cardiovascular Disease, 56 rue Leblanc, Paris 75015 France; b Schiller Médical SAS, 4 rue L. Pasteur 67160 Wissembourg, France

**Keywords:** Paediatric out-of-hospital cardiac arrest, Automated external defibrillator, Shock efficacy, Shock advisory system, Survival, Energy dose

## Abstract

**Objective:**

This study aims to quantify the reliability of automated external defibrillators (AED) in paediatric out-of-hospital cardiac arrests (pOHCA) by evaluating the defibrillation and the shock advisory system efficacy. Furthermore, the relationship between the initial energy dose and patient outcomes is analysed.

**Methods:**

We studied data from all pOHCA cases (age < 18 years) treated by the Paris Fire Brigade between January 2010 and December 2018, limited to those with available AED signals. The efficacy of shocks is the primary outcome. The secondary outcomes are the shock advisory system performance, pre-hospital return of a spontaneous circulation (ROSC), survival and energy dose. Energy dose, weight and age are compared using a Wilcoxon test according to the outcome’s values.

**Results:**

A total of 1,990 electrocardiogram strips extracted from 349 pOHCA cases were included in the study. Shock advisory system had a sensitivity of 89.4% and a specificity of 99.8% for the detection of shockable rhythms. Shock efficacy observed for all patients who received a shock was 83.1% and first shock efficacy for patients in initial ventricular fibrillation was 96%. Patients who received a shock had a pre-hospital ROSC rate of 74.3%, a survival rate at hospital admission of 71.4% and 34.3% at hospital discharge.

**Conclusion:**

This study shows that AED detect shockable rhythm with a good sensitivity and specificity and that shocks are associated with a very high rates of termination of shockable rhythms in pOHCA.

## Introduction

Paediatric out-of-hospital cardiac arrest (pOHCA) is a family disaster and still occurs at a rate of 4 to 9 per 100,000 people annually in industrialized countries.^1^, ^2^ Since 1979, Automated External Defibrillators (AED) have been instrumental in providing critical care during such emergencies. Initially tailored for adults, AED analyse cardiac rhythms and deliver an electric shock to restore spontaneous circulation in cases of ventricular fibrillation (VF) or rapid ventricular tachycardia (VT). The International Liaison Committee on Resuscitation (ILCOR), the American Heart Association (AHA) and the European Resuscitation Council (ERC), have recommended and approved AED use for all children.^3^, ^4^, ^5^

A key consideration in paediatric use of AED is the efficacy of shock and the adjustment of energy doses.^6^ ILCOR recommends an initial energy dose of 2–4 J/kg and subsequent doses can be increased up to 10 J/kg.^7^

Another key consideration is the shock advisory system that must be assessed using paediatric arrhythmia signals.^8^ Studies have demonstrated reliable accuracy in rhythm detection when tested on paediatric rhythms obtained mainly from in-hospital cardiac arrest (IHCA) cases.^9^, ^10^, ^11^, ^12^, ^13^ However, these devices must be rigorously tested in paediatric settings to ensure their reliability and efficacy.

This study aims to bridge the knowledge gaps in paediatric defibrillation regarding defibrillation efficacy, rhythm detection, survival, and optimal energy dose. This study will evaluate the reliability of AED used in Paris by the Paris Fire Brigade. It will also evaluate patient outcomes. This observational study is a prerequisite to a future improvement of paediatric defibrillation practices, ultimately aiming at enhancing survival rates and clinical outcomes in paediatric emergency care.

## Materials and methods

### Study design

This is an observational study, reviewing all the paediatric cardiac arrests (age < 18 years) treated by Paris Fire Brigade from January 2010 to December 2018. The firefighters serve Paris and its suburbs, which represent a population of 6,754,300 people including 1,250,000 children under 14 years (INSEE 2014).^14^, ^15^, ^16^, ^17^

The study features a two-tiered physician-manned emergency system. Firefighter's Basic Life Support teams are the first responders. They manage pOHCA according to the current European Resuscitation Council guidelines.^18^ Advance Life Support teams follow within minutes and provide medical care on the field.

The firefighters use AED (DGT7® or Fred Easy®, Schiller Medical, Wissembourg, France) that record events during resuscitation including electrocardiograms (ECG) and transthoracic impedance signals. Both DGT7® and Fred Easy® deliver a biphasic truncated exponential waveform with a pulsed energy (Multipulse Biowave®).

Two types of defibrillation pads are available: adult and child pads. Child pads are applied on patients under 8 years old or weighing less than 25 kg. The patient weight was estimated by the BLS team or measured at the hospital, if the patient was admitted. The energy delivered with child pads is 30 J for the first shock and 50 J for the other, while with adult pads it is 150 J for the first shock and 200 J for the others.

The epidemiologic and survival data of the patients were collected prospectively and extensively by the Paris Sudden Death Expertise Centre. All pOHCA data in Paris and its suburbs are provided by the hospitals and recorded according to Utstein registry.^19^ The appropriate review boards approved the study (CNIL approval #912309 and CCTIRS approval #12336).

### Data annotation

The ECG strips (i.e. AED analysis/shock periods) were annotated in 2 categories: shockable and non-shockable rhythm. The annotated rhythms are VF, fine-VF and rapid-VT for shockable rhythm; asystole, normal sinus rhythm, and other non-shockable rhythm for non-shockable rhythm. If the first annotation is VF, the pOHCA is categorized as initial VF (iVF) patient, else it is categorized as non-iVF patient. Pre-shock rhythms were annotated 5 s before the shock as either shockable or non-shockable rhythm. Post-shock rhythms were annotated at 5 s, 15 s, 30 s, 60 s and 120 s after shock as organized rhythm, VF or asystole. Time to refibrillation (recurrence of VF) was measured. Additionally, return of organized rhythm (ROOR) is annotated at 60 s post-shock.^20^ The presence of artifacts or pacemaker were notified.

Two emergency physicians (DJ, BF) with experience in cardiac arrests cardiology from Paris Fire Brigade and one cardiologist (XJ) from Paris Sudden Death Expertise Centre visually revised 10 s ECG strip (lead II) and associated transthoracic impedance. Their annotations were made blinded to the outcomes. A consensus decision was required in cases of annotation disagreement. Without consensus, the rhythm was annotated as undefined and excluded. ECG strips with a rhythm transition or with an analysis interrupted by chest compressions were also excluded.

Inclusions are specified at the end of each analysis paragraph below.

### Analysis

#### Primary outcome: Shock efficacy

The shock efficacy is defined as the cessation of shockable rhythm 5 s after shock delivery whether rhythm is asystole, pulseless electrical activity or organized rhythm. AHA and ERC do not provide target efficacy for shocks. We chose a target efficacy of 80% for first shock of iVF patients (see supp file Goal Justification).^21^, ^22^, ^23^, ^24^, ^25^, ^26^, ^27^, ^28^, ^29^, ^30^

Refibrillation appearing less than 5 s after the shock is defined as persistent VF. Analyses of refibrillation and persistent VF provide complementary insights into the primary outcome.

The ECG strips included in the shock efficacy analysis were those corresponding to an AED analysis with shock advice, where a shock was delivered and the pre-shock rhythm was annotated as shockable.

#### Secondary outcomes

##### Shock advisory system

Shock advisory system performance was evaluated for both sensitivity and specificity. Currently, all the recommendations concern general out-of-hospital cardiac arrest (OHCA), and no rules are yet established specifically for paediatrics. By default, we used those for the general population. The performance goals recommended in absence of artifact and pacemaker by Kerber *al.* and the International Electrotechnical Commission standard are described in supplementary file (Table A).^5^, ^31^ They chose the one-sided 90% lower confidence limit as the threshold for statistical significancy.

The ECG strips included in the shock advisory system assessment were those without artifact and without pacemaker.

##### Survival rate

The defibrillation shock should be able to increase the chance of survival. We evaluated the survival rate on the outcomes: prehospital return of spontaneous circulation (ROSC), survival at hospital admission and survival at hospital discharge.

The pOHCA patients included in the analysis of survival were those for whom the AED recommended a shock, a shock was delivered, the pre-shock rhythm was annotated as shockable, and patient data was available.

##### Energy dose

We compared the energy dose, weight and age between patients with favourable and poor outcomes. The outcomes were the shock success, ROOR at 60 s post-shock, prehospital ROSC, survival at hospital admission and survival at hospital discharge. For both pad types (child/adult), the energy delivered at the first shock was compared according to the outcome’s value (failure or success). A similar analysis was conducted considering all shocks. For prehospital ROSC, survival at hospital admission and survival at hospital discharge that may occur after multiple shocks, the median energy dose delivered during the intervention was used.

The ECG strips included in the energy dose analysis were those from iVF patients corresponding to AED analyses with shock advice, where a shock was delivered, the pre-shock rhythm was annotated as shockable and patient data was available.

All statistics are conducted using R (Version 3.6.2). When the data are normally distributed, a *t*-test is used, otherwise a Wilcoxon test is used.

## Results

**Fig. 1:**
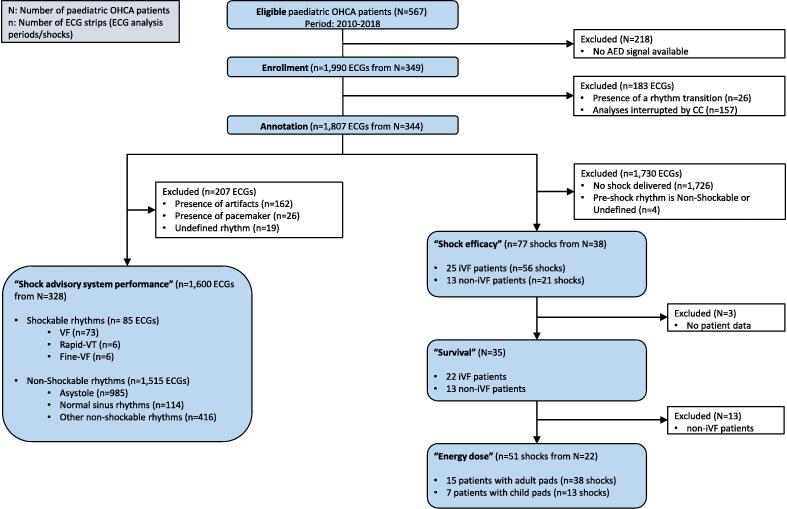
Flow chart of pOHCA shock and rhythm analysis (with n the number of strips and N the number of patients). OHCA: Out-of-hospital cardiac arrest; AED: Automated External Defibrillator; ECG: Electrocardiogram; CC: Chest Compressions; VF: Ventricular Fibrillation; VT: Ventricular Tachycardia; iVF: initial VF.

All the pOHCA that occurred between January 2010 and December 2018 were recorded. In the study, 1,990 ECG strips extracted from 349 pOHCA were included. The flow chart in Fig. 1 describes the ECG strips and corresponding pOHCA included: 77 strips (38 pOHCA) were eligible for shock efficacy survey and 1,600 strips (328 pOHCA) for shock advisory system performance survey. Three patients did not have available medical data, thus the survival analysis have been limited to 35 patients including 22 iVF patients. The energy dose analysis focused on the 22 iVF patients and their 51 strips. Due to age disparity, different types of pads were used. In our cohort, 60.0% (21/35) were treated with adult pads and 40.0% (14/35) with child pads. The baseline characteristics of the patients are described in Table 1.

**Table 1:** Baseline characteristics of all 349 patients and 38 patients who received a shock with electrical signal available. Column Value represents the patients with available data.

	**All patients (N = 349)**		**Patients with shock (N = 38)**	
**Characteristics**	**Value**	**Missing data**	**Value**	**Missing data**
**Male**, *n (%)*	168 (56.4%)	51	20 (58.8%)	4
**Age (years)**, *median [IQR]*	5.18 [0.74–13.8]	50	12.4 [8.71–15.6]	2
**Weight (kg)**, *median [IQR]*	12.0 [6.57–30.0]	192	33.7 [26.3–43.6]	2
**Arrest during sport**, *n (%)*	11 (3.69%)	51	7 (20.6%)	4
**Initial VF**, *n (%)*	31 (9.09%)	8	25 (65.8%)	0
**Shock delivered**, *n (%)*	43 (14.7%)	57	38 (100%)	0
**Public location**, *n (%)*	87 (29.3%)	52	17 (50.0%)	4
**Witnessed OHCA**, *n (%)*	145 (49.0%)	53	28 (82.4%)	4
**Bystander CPR**, *n (%)*	74 (25.0%)	53	16 (47.1%)	4
**Prehospital ROSC**, *n (%)*	91 (31.4%)	59	26 (74.3%)	3
**Survival at hospital admission**, *n (%)*	82 (43.6%)	161	25 (71.4%)	3
**Survival at hospital discharge**, *n (%)*	21 (7.22%)	58	12 (34.3%)	3

VF: Ventricular Fibrillation; OHCA: Out-of-hospital Cardiac Arrest; CPR: Cardiopulmonary resuscitation; ROSC: Return of a Spontaneous Circulation.

### Primary outcome: Shock efficacy

The global efficacy of the first shock for all patients was 89.5% (34/38) (Table 2A). The global shock efficacy observed for all patients was 83.1% (64/77) (Table 2B). Among 38 patients who received a shock, iVF patients represented 65.8% (25/38). The first shock efficacy for iVF patients was 96% (24/25). Finally, the first shock efficacy for non-iVF patients was more limited with 76.9% (10/13).

**Table 2:** Shock efficacy and refibrillation for first shocks in panel (A) and all shocks in panel (B).

A.				
		**All patients with shock**	**iVF patients**	**Non-iVF patients**
**First Shock**	N	38	25	13
	Successful	34 (89.5%)	24 (96.0%)	10 (76.9%)
	Unsuccessful	4 (10.5%)	1 (4.0%)	3 (23.1%)
Refibrillation	N	38	25	13
	Persistent VF	4 (10.5%)	1 (4%)	3 (23.1%)
	Refibrillation	12 (31.6%)	9 (36%)	3 (23.1%)
	No refibrillation	21 (55.3%)	15 (60%)	6 (46.1%)
	Undefined	1 (2.6%)	0 (0%)	1 (7.7%)
Time to refibrillation (s)	N	12	9	3
	Median	24.75	25.00	10.90
	[IQR]	[15.33–35.47]	[20.10–37.50]	[8.70–21.60]

B.				
		**All patients with shock**	**iVF patients**	**Non-iVF patients**
**All Shock**	N	77	56	21
	Successful	64 (83.1%)	46 (82.1%)	18 (85.7%)
	Unsuccessful	13 (16.9%)	10 (17.9%)	3 (14.3%)
Refibrillation	N	77	56	21
	Persistent VF	13 (16.9%)	10 (17.9%)	3 (14.3%)
	Refibrillation	26 (33.8%)	18 (32.1%)	8 (38.1%)
	No refibrillation	36 (46.7%)	28 (50.0%)	8 (38.1%)
	Undefined	2 (2.6%)	0 (0.0%)	2 (9.5%)
Time to refibrillation (s)	N	26	18	8
	Median	20.50	24.15	9.75
	[IQR]	[11.15 – 30.65]	[20.18–34.47]	[6.18–15.95]

VF: Ventricular Fibrillation; iVF: initial VF.

**Fig. 2:**
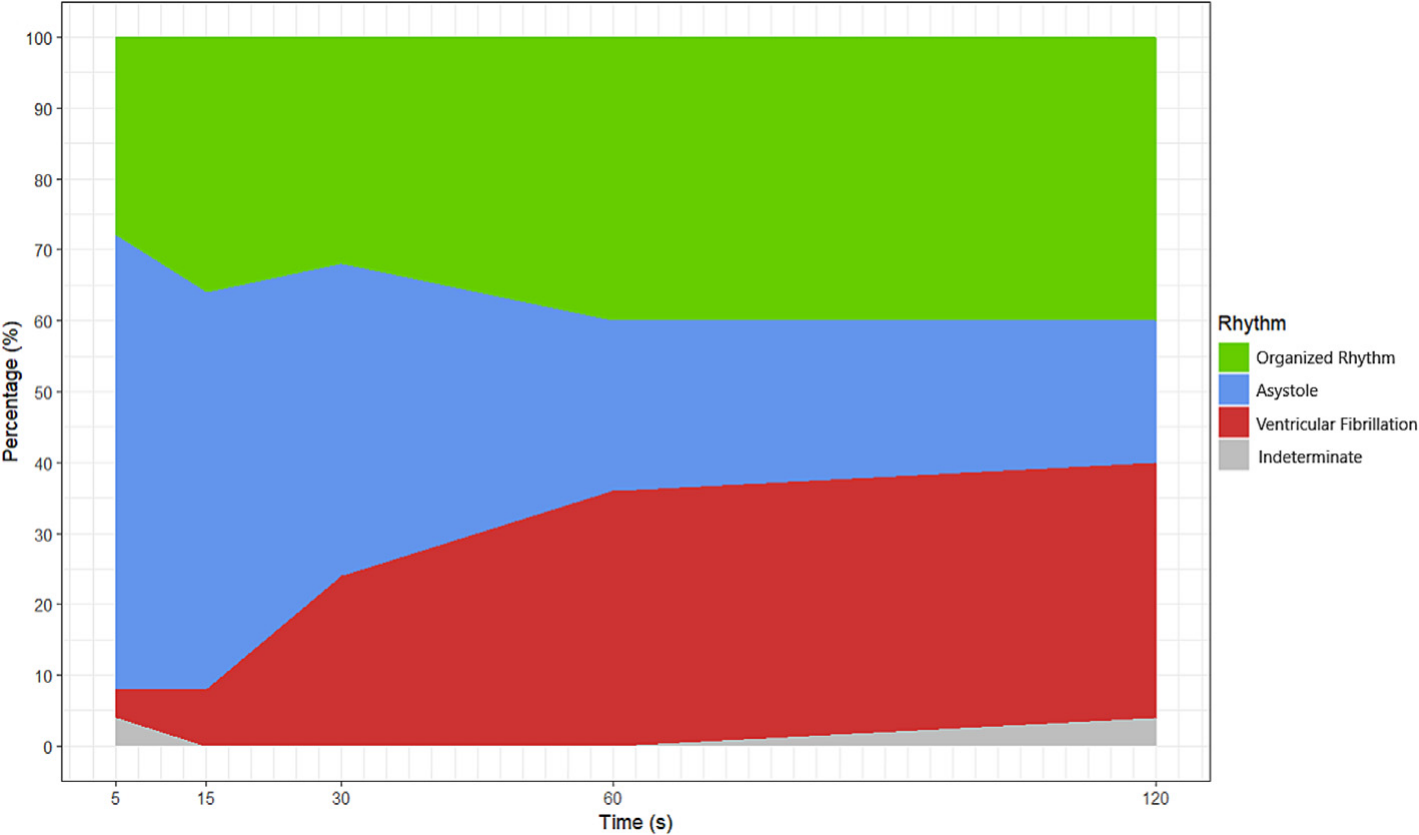
Rhythm in the first two minutes after the first shock of iVF patients. Rhythm annotations were done at 5, 15, 30, 60 and 120 s after the first shock. Annotations before 5 s was not possible because of shock artifacts and unstable electrocardiographic baseline. Indeterminate: unreadable post-shock rhythm due to presence of chest compressions.

Fig. 2 represents the rhythm in the first two minutes after the first shock of iVF patients. 36% of the first shocks of iVF patients were followed by a refibrillation beyond 5 s post-shock (Table 2).

### Secondary outcomes

#### Shock advisory system

We analysed 1,600 ECG strips among which 85 annotated shockable and 1,515 annotated non-shockable (Fig. 1). VF was the most frequent shockable rhythm whereas asystole was the most frequent non-shockable rhythm. Table 3 summarizes the annotated rhythms and the performances of the shock advisory system.

**Table 3:** Sensitivity (Se) and specificity (Sp) considering artifact free ECG strips according to Kerber and standard recommendations.^5^

**Rhythm**	**Sample Size**	**Shock advised**	**No shock advised**	**Performances (Se/Sp)**	**90% 1-sided LCL**
VF	73	71	2	97%	94%
Fine VF	6	3	3	50%*	*
Rapid-VT	6	2	4	33%*	*
Asystole	985	0	985	100%	100%
Normal Sinus Rhythm	114	0	114	100%	100%
Other Non-shockable Rhythm	416	3	413	99%	98%

Note: *due to the small size of this subgroup, no measure of dispersion.

VF: Ventricular Fibrillation; VT: Ventricular Tachycardia; LCL: Lower Confidence Limit.

Among 85 shockable rhythms, 9 (10.6%) rhythms were wrongly detected as non-shockable by the algorithm. The 9 rhythms were from 7 different patients. Of them, five achieved a prehospital ROSC and survived hospital admission, while one did not. Two survived at hospital discharge whereas four did not. Data is missing for one patient.

Among 1,515 non-shockable rhythms, 3 (0.2%) rhythms were wrongly detected as shockable by the algorithm. The 3 rhythms were from 3 different patients. Two achieved a prehospital ROSC and survived hospital admission, and one survived at hospital discharge.

#### Survival

Over the whole paediatric population (N = 349), 91/290 patients achieved a prehospital ROSC (31.4%). The survival rate at hospital admission was 43.6% (82/188) and the survival rate at hospital discharge was 7.22% (21/291) (Table 1).

In this cohort, 38 patients with a shockable rhythm were shocked and patient data were available for 35. Among them, 26 achieved a prehospital ROSC (74.3%). The survival rate at hospital admission was 71.4% (25/35) and the survival rate at hospital discharge was 34.3% (12/35) (Table 1). The survival rate at hospital discharge for non-shockable patients was 3.52% (9/256).

Among 22 iVF patients, 17 (77.3%) achieved prehospital ROSC. The survival rate at hospital admission was 72.7% (16/22) and the survival rate at hospital discharge was 40.9% (9/22).

Among 13 non-iVF patients, 9 (69.2%) achieved a prehospital ROSC. The survival rate at hospital admission was 69.2% (9/13) and the survival rate at hospital discharge is 23.1% (3/13).

#### Energy dose

Among the 35 patients, 22 were iVF patients. Regarding child pads, the delivered energy dose of first shock was lower for any of the following outcomes in case of success (Table 4A), but the differences did not reach statistical significancy: ROOR at 60 s post-shock (1.11 [0.97–1.55] vs 4.19 [3.74–4.63] J/kg, p = 0.095), prehospital ROSC (1.33 [1.00–2.17] vs 5.08 J/kg), survival at hospital admission (1.11 [0.97–1.55] vs 4.19 [3.74–4.63] J/kg, p = 0.095) and survival at hospital discharge (1.04 [1.00–1.07] vs 2.38 [1.55–3.30] J/kg, p = 0.381). For patients who had child pads applied, those with ROOR at 60 s post-shock and survival had higher weight (28.0 [20.0–30.0] vs 7.35 [6.63–8.07] kg, p = 0.079) and age (8.73 [8.66–10.0] vs 0.33 [0.30–0.36] years, p = 0.095) compared with those with no ROOR at 60 s post-shock or survival but the differences did not reach statistical significancy (Table 4B, C). Similar results were found considering all shocks of iVF patients (Table B).

**Table 4:** Comparison of energy doses (J/kg) on panel (A), weights (kg) on panel (B) and ages (years) on panel (C) using a Wilcoxon test for each outcomes considering first shocks of initial VF patients. Median [IQR] dose for failure and success of each outcome is presented.

A.						
	**Dose with adult pads (J/kg)**			**Dose with child pads (J/kg)**		
**Outcomes**	**Failure**	**Success**	**p-val**	**Failure**	**Success**	**p-val**
Shock success	(N = 0)	3.52 [2.61–4.24] (N = 15)	−	(N = 0)	1.55 [1.04–2.84] (N = 7)	−
ROOR at 60 s	3.72 [2.42–4.50] (N = 8)	3.52 [3.05–3.74] (N = 7)	0.955	4.19 [3.74–4.63] (N = 2)	1.11 [0.97–1.55] (N = 5)	0.095
ROSC	4.24 [3.84–5.26] (N = 4)	3.47 [2.55–3.74] (N = 11)	0.104	5.08 (N = 1)	1.33 [1.00–2.17] (N = 6)	−
Survival at hospital admission	3.88 [3.70–4.85] (N = 4)	3.47 [2.55–4.10] (N = 11)	0.226	4.19 [3.74–4.63] (N = 2)	1.11 [0.97–1.55] (N = 5)	0.095
Survival at hospital discharge	3.65 [3.24–4.11] (N = 8)	3.47 [2.58–4.10] (N = 7)	0.613	2.38 [1.55–3.30] (N = 5)	1.04 [1.00–1.07] (N = 2)	0.381

B.						
	**Weight of patients with adult pads (kg)**			**Weight of patients with child pads (kg)**		
**Outcomes**	**Failure**	**Success**	**p-val**	**Failure**	**Success**	**p-val**
Shock success	(N=0)	41.9 [33.7–51.0] (N=15)	−	(N=0)	20.0 [10.7–29.0] (N=7)	−
ROOR at 60s	39.8 [30.1–48.8] (N=8)	43.5 [40.9–50.5] (N=7)	0.487	7.35 [6.63–8.07] (N=2)	28.0 [20.0–30.0] (N=5)	0.079
ROSC	36.5 [30.1–40.6] (N=4)	43.5 [37.0–58.5] (N=11)	0.214	5.91 (N=1)	24.0 [14.4–29.5] (N=6)	−
Survival at hospital admission	39.7 [34.6–41.0] (N=4)	43.5 [33.7–58.5] (N=11)	0.396	7.35 [6.63–8.07] (N=2)	28.0 [20.0–30.0] (N=5)	0.079
Survival at hospital discharge	39.7 [30.1–43.6] (N=8)	45.0 [37.9–58.5] (N=7)	0.203	12.6 [8.79–20.0] (N=5)	29.0 [28.5–29.5] (N=2)	0.241

C.						
	**Age of patients with adult pads (years)**			**Age of patients with child pads (years)**		
**Outcomes**	**Failure**	**Success**	**p-val**	**Failure**	**Success**	**p-val**
Shock success	(N=0)	14.0 [11.7–15.9] (N=15)	−	(N=0)	8.66 [1.35–9.38] (N=7)	−
ROOR at 60s	13.1 [8.57–14.1] (N=8)	16.0 [14.6–16.7] (N=7)	0.029	0.33 [0.30–0.36] (N=2)	8.73 [8.66–10.0] (N=5)	0.095
ROSC	14.2 [11.1–15.9] (N=4)	14.0 [12.1–15.7] (N=11)	0.949	0.27 (N=1)	8.69 [3.89–9.71] (N=6)	−
Survival at hospital admission	15.9 [13.3–16.1] (N=4)	13.9 [11.7–15.0] (N=11)	0.489	0.33 [0.30–0.36] (N=2)	8.73 [8.66–10.0] (N=5)	0.095
Survival at hospital discharge	14.9 [11.1–16.1] (N=8)	13.9 [12.1–15.0] (N=7)	0.779	2.30 [0.40–8.66] (N=5)	9.38 [9.06–9.71] (N=2)	0.381

ROOR: Return of Organized Rhythm; ROSC: Return of Spontaneous Circulation; IQR: Interquartile Range.

No difference was observed in energy dose for adult pads between failure and success. No test was carried out on shock success and prehospital ROSC due to a too small sample size.

## Discussion

The aim of this study was to evaluate the reliability of AED in paediatric use. The results showed effectiveness of defibrillation shocks using Fred Easy® and DGT7® defibrillators. The shock advisory system exceeded recommendations for specificity and sensitivity in all but one category (rapid-VT). In addition, the survival rate is higher for patients with shockable rhythms than for those with non-shockable rhythms. Although no statistically significant differences were reached, among patients with child pads, a trend suggests that better outcomes are associated with lower shock doses as well as greater weight and age.

### Primary outcome: Shock efficacy

Shock efficacy target of 80% was reached for first shocks delivered to iVF patients (96.0%). Efficacy of first shocks delivered to non-iVF patients was 76.9%. Concerning all shocks delivered to both iVF and non-iVF patients, similar efficacies were found, respectively 82.1% and 85.7%. We observed an earlier refibrillation for non-iVF patients compared to iVF patients (p = 0.011).

A few studies suggest that resumption of chest compressions is associated with refibrillation.^32^, ^33^ Despite satisfactory efficacy results, 33.8% of shocks were followed by a refibrillation beyond 5 s post-shock (Table 2). However, the proportion of refibrillation in our study is much lower than what is typically found in the literature for adults (61%).^34^ As cardiac causes are more likely to lead to refibrillation, the difference in aetiology, with a higher proportion of non-medical and non-cardiac causes in paediatric patients, may explain the lower refibrillation rates compared to adults.^35^, ^36^.

### Secondary outcomes

#### Shock advisory system

Specificity for non-shockable rhythms was 100% for normal sinus rhythm and over 99% for other rhythms. Sensitivity for VF exceeded the recommended levels (94%). However, the minimum sample size was not always met, with only 73 VF cases, 6 rapid-VT and 6 fine-VF. Since all guidelines pertain to general OHCA and no specific rules exist for paediatric cases, we followed the recommendations for the general population.^5^, ^31^

From 7 different patients, 9 false negatives were detected. The possible reasons are 1) heart rate just under the algorithm VT threshold (150 bpm) for shock/no-shock decision, 2) very high heart rate for VF (>500 bpm) which is akin to myogram noise, 3) wide ventricular complex supraventricular tachycardia that was considered shockable by the annotators and 4) fine-VF.

False positives were detected in 3 strips, from 3 different patients that present very large T-wave amplitudes that lead to double counting of the complexes.

Rhythms that result in false positives and false negatives detections are more frequent in pOHCA. However, the sensitivity and specificity of the shock advisory system are comparable to those observed in adult OHCA. Krasteva *et al.* also noted that the differences between paediatric and adult rhythms do not necessarily cause a decrease in shock advisory system accuracy.^13^

#### Survival

The survival rates are influenced by factors such as initial rhythm, duration of pulselessness, emergency response time, presence of witness, bystander cardiopulmonary resuscitation and quality of post-resuscitation care.^37^

Numerous initiatives enhanced survival rates for paediatric IHCA, with an increase from 14% to 43% over a decade, without a corresponding rise in severe disability among the survivors.^38^ While statistics from Australia/New Zealand, Denmark, and North America did not show enhanced survival rates of pOHCA, recent figures from Sweden reveal a positive trend: the 30-day survival rate for children aged 0–21 years climbed from 6% during 1992–1998 to 14% between 2007 and 2012.^1^, ^39^, ^40^, ^41^

In our study, we observed a survival rate at hospital discharge of 36.1% for shockable patients, against 3.52% for non-shockable patients. Among shockable patients, survival rate at hospital discharge is 40.9% for iVF patients and 28.6% for non-iVF patients. These results highlight the importance of early VF/VT recognition and are in accordance with those mentioned by Atkins and Kenney who reported a survival of 38%.^42^

#### Energy dose

For iVF patients with child pads, we observed that successful outcomes are possibly related to lower energy dose, although the differences did not reach statistical significancy. For adult pads, we did not observe any difference in energy dose between failure and success.

Our results suggest a trend indicating that patients with ROOR at 60 s post-shock may have been treated with a lower energy dose compared to those without ROOR at 60 s post-shock, although this difference did not reach statistical significance. The same trend was found for survival at hospital admission. These results are consistent with Hoyme *et al.* who associated higher survival at hospital discharge with an energy dose range of 1.7–2.5 J/kg.^43^ Unfortunately, deeper analysis is limited by the small number of patients (N = 7) in our study. The lack of data was also the main limitation of the other studies that focused on both IHCA and OHCA or only on small sample size OHCA.^44^, ^45^, ^46^, ^47^

As our study concerns AED, the delivered energy is identical for every patient with child pads (30 J), thus the delivered energy dose reflects the patient's weight. Moreover, for paediatric patients, weight is strongly correlated with age. Consequently, if an outcome is related to lower doses, it is not possible to conclude that it is higher age, lower doses or both that is associated with the outcome.

## Study limitations

We did not reach the AHA sample size targets for shockable rhythms that are difficult to reach for paediatric patients. Indeed, during the current study in Paris and its suburbs (over 6.5 million people), 349 pOHCA were included in 9 years: 38 were shocked (10.9%), 25 were iVF patients (7.16%). Which leads to the estimation of about 4 shockable pOHCA managed by Paris Fire Brigade per year.

In the same context, about 400 shockable OHCA was managed by Paris Fire Brigade per year from January 2010 to January 2014.^32^

Compared to adults, paediatric VF/VT is rare as occurrence of shockable pOHCA is 1% (4/400) of occurrence of shockable OHCA.

One limitation of our study is the decision to exclude all ECG strips presenting artifacts (Fig. 1). An alternative approach would have been to include strips where it was possible for a human to analyse the rhythm. Incorporating such rhythms and challenging cases could have provided a more representative picture of AED performance in a clinical setting, accounting for the obstacles that complicate analysis for both humans and machines. However, as numerous of these ECG strips are unreadable and therefore non-annotable, we chose to exclude them all to avoid introducing selection bias, even at the expense of reduced statistical significancy. This choice of exclusion criteria also facilitates reproducibility of the study.

Another key limitation of this study is the absence of data on neurological outcomes and long-term survival, which restricts our ability to fully assess patient recovery.

Additionally, the study only evaluated one type of AED, raising questions about the generalizability of the results to other AED brands. Further research is needed to determine whether similar outcomes would be observed with different devices.

Finally, some degree of information bias regarding weight cannot be ruled out, as it was estimated by BLS teams in certain cases.

## Conclusions

This study shows that shocks from one type of AED are associated with a very high rates of termination of shockable rhythms in pOHCA. The efficiency of defibrillation shocks is higher for the first shock of iVF patients, reaching more than 95%, using a pulsed biphasic truncated exponential waveform.

The study also shows that the AED detect shockable rhythm with a good sensitivity and specificity. The shock advisory system dedicated to diagnosing paediatric patient rhythms met the sensitivity and specificity criteria required by the AHA for general population.

## CRediT authorship contribution statement

**Emma Menant:** Writing – review & editing, Writing – original draft, Visualization, Validation, Formal analysis, Data curation. **Delphine Lavignasse:** Writing – review & editing, Validation, Software, Resources, Methodology, Investigation, Formal analysis, Data curation, Conceptualization. **Sarah Ménétré:** Writing – review & editing, Validation, Supervision, Software, Resources, Methodology, Formal analysis, Conceptualization. **Jean-Philippe Didon:** Writing – review & editing, Supervision, Conceptualization. **Xavier Jouven:** Writing – review & editing, Supervision, Project administration, Funding acquisition, Formal analysis, Conceptualization.

## Funding

This study did not receive any specific grant from funding agencies in the public, commercial, or not-for-profit sectors, but was a part of a thesis funded by a grant.

## Declaration of competing interest

The authors declare the following financial interests/personal relationships which may be considered as potential competing interests: Emma Menant reports a relationship with SCHILLER Médical, Wissembourg, France that includes: receiving research support. Delphine Lavignasse reports a relationship with SCHILLER Médical, Wissembourg, France that includes: receiving research support. Sarah Ménétré reports a relationship with SCHILLER Médical, Wissembourg, France that includes: employment. Jean-Philippe Didon reports a relationship with SCHILLER Médical, Wissembourg, France that includes: employment. Xavier Jouven reports a relationship with SCHILLER Médical, Wissembourg, France that includes: receiving funds for scientific consulting. If there are other authors, they declare that they have no known competing financial interests or personal relationships that could have appeared to influence the work reported in this paper.

## Glossary

AED: Automated External Defibrillator
AHA: American Heart Association
CPR: Cardiopulmonary Resuscitation
ECG: Electrocardiogram
ERC: European Resuscitation Council
IHCA: In-Hospital Cardiac Arrest
ILCOR: International Liaison Committee on Resuscitation
IQR: Interquartile Range
iVF: initial Ventricular Fibrillation
OHCA: Out-of-Hospital Cardiac Arrest
pOHCA: pediatric Out-of-Hospital Cardiac Arrest
ROOR: Return Of Organized Rhythm
ROSC: Return Of Spontaneous Circulation
Se: Sensitivity
Sp: Specificity
VF: Ventricular Fibrillation
VT: Ventricular Tachycardia
