# Supplementary material for: Automated external defibrillator: Rhythm analysis and defibrillation on paediatric out-of-hospital cardiac arrest
Source: Resusc Plus. 2025 Jan 16;22:100873. doi: 10.1016/j.resplu.2025.100873 (PMC11803253; doi:10.1016/j.resplu.2025.100873)
Supplement: Supplementary Data 1 [file mmc1.docx]

# **Supplementary material**

**Automated external defibrillator: rhythm analysis and defibrillation on paediatric out-of-hospital cardiac arrests**

Emma Menant^a^, Delphine Lavignasse^a^, Sarah Ménétré^b^, Jean-Philippe Didon^b^, Xavier Jouven^a^

^a^Université Paris Cité, INSERM U970, Paris Cardiovascular Research Centre (PARCC), Integrative Epidemiology of Cardiovascular Disease, 56 rue Leblanc, Paris 75015, France

^b^ Schiller Médical SAS, 4 rue L. Pasteur, 67160 Wissembourg, France

**Table A**. Rhythm definitions, sample size and performance goal recommendations for the different ECG categories in absence of artifacts and pacemaker.

Note: *90% one-sided lower confidence limit (LCL90%) indicates if the sensitivity(Se)/specificity(Sp) have a low enough disparity in accordance with the test sample size.

| **Rhythm** | | | **Sample Size goal** | **Performance goal** | |
| --- | --- | --- | --- | --- | --- |
|  | **Type** | **Description** |  | **Se/Sp** | **LCL90%*** |
| Non shockable | NSR | Normal Sinus Rhythm (P-QRS-T is visible) with heart rate (>40 bpm and < 100 bpm) | 100 | 99% | >97% |
|  | ONR | Other non-shockable rhythms: Atrial Fibrillation/Flutter, sinus bradycardia, Supraventricular Tachycardia, Premature Ventricular Contractions, Heart Blocks… | 30 | 95% | >88% |
|  | Asystole | Asystole (peak-to-peak amplitude <= 100µV during more than 4 seconds | 100 | 95% | >92% |
| Shockable | VF | Coarse VF (peak-to-peak amplitude > 200µV) | 200 | 90% | >87% |
|  | rapid-VT | Rapid VT (heart rate >= 150min^-1^), VT rushes that last more than 8 seconds among the annotated segment | 50 | 75% | >67% |
| Intermediate | slow-VT | Slow VT (< 150min^-1^) | Report only | Report only | Report only |
|  | fine-VF | Fine VF (peak-to-peak amplitude > 100µV and ≤ 200µV) | Report only | Report only | Report only |

**Table B.** Comparison of energy doses (J/kg) on panel (A), weights (kg) on panel (B) and ages (years) on panel (C) using a Wilcoxon test for each outcomes considering all shocks of initial VF patients. Median [IQR] dose for failure and success of each outcomes are presented.

Note: *As ROSC and survival can happened after several shocks, the median delivered dose of each initial VF patients are considered.

ROOR: Return of Organized Rhythm; ROSC: Return of Spontaneous Circulation; IQR : Interquartile Range

1.

|  | **Energy dose with adult pads (J/kg)** | | | **Energy dose with child pads (J/kg)** | | |
| --- | --- | --- | --- | --- | --- | --- |
| **Outcomes** | **Failure** | **Success** | **p-val** | **Failure** | **Success** | **p-val** |
| Shock success | 3.95 [3.90-3.97]  (N=9) | 3.70 [3.45-4.00]  (N=29) | 0.099 | (N=0) | 2.30 [1.11-3.30]  (N=13) | - |
| ROOR at 60s | 3.94 [3.96-3.98]  (N=20) | 3.70 [3.46-3.89]  (N=18) | 0.174 | 4.19 [3.12-5.08]  (N=4) | 1.40 [0.97-2.30]  (N=9) | 0.013 |
| ROSC* | 4.23 [3.82-5.28]  (N=4) | 3.47 [2.55-3.74]  (N=11) | 0.104 | 5.08  (N=1) | 1.59 [1.04-2.28]  (N=6) | - |
| Survival at hospital admission* | 3.87 [3.71-4.86]  (N=4) | 3.47 [2.55-4.09]  (N=11) | 0.226 | 4.19 [3.74- 4.63]  (N=2) | 1.11 [1.02-2.08]  (N=5) | 0.095 |
| Survival at hospital discharge* | 3.65 [3.24-4.09]  (N=8) | 3.47 [2.58-4.09]  (N=7) | 0.694 | 2.34 [2.08- 3.30]  (N=5) | 1.04 [1.00-1.07]  (N=2) | 0.190 |

2.

|  | **Weight of patients with adult pads (kg)** | | | **Weight of patients with child pads (kg)** | | |
| --- | --- | --- | --- | --- | --- | --- |
| **Outcomes** | **Failure** | **Success** | **p-val** | **Failure** | **Success** | **p-val** |
| Shock success | 39.5 [39.5-39.5]  (N=9) | 41.9 [34.0-45.0]  (N=32) | 0.120 | (N=0) | 20.0 [8.79-30.0]  (N=13) | - |
| ROOR at 60s | 39.5 [39.1-40.1]  (N=20) | 41.9 [39.6-44.8]  (N=18) | 0.048 | 7.35 [5.91-11.6]  (N=4) | 28.0 [12.6-30.0]  (N=9) | 0.034 |
| ROSC* | 36.5 [30.1-40.6]  (N=4) | 43.5 [37.0-58.5]  (N=11) | 0.214 | 5.91  (N=1) | 24.0 [14.4-29.5]  (N=6) | - |
| Survival at hospital admission* | 39.7 [34.6-41.0]  (N=4) | 43.5 [33.7-58.5]  (N=11) | 0.396 | 7.35 [6.63-8.07]  (N=2) | 28.0 [20.0-30.0]  (N=5) | 0.079 |
| Survival at hospital discharge* | 39.7 [30.1-43.6]  (N=8) | 45.0 [37.9-58.5]  (N=7) | 0.203 | 12.6 [8.79-20.0]  (N=5) | 29.0 [28.5-29.5]  (N=2) | 0.241 |

3.

|  | **Age of patients with** **adult pads (years)** | | | **Age of patients with** **child pads (years)** | | |
| --- | --- | --- | --- | --- | --- | --- |
| **Outcomes** | **Failure** | **Success** | **p-val** | **Failure** | **Success** | **p-val** |
| Shock success | 15.8 [15.8-15.8]  (N=9) | 14.6 [12.7-16.0]  (N=29) | 0.354 | (N=0) | 8.66 [0.40-8.73]  (N=13) | - |
| ROOR at 60s | 15.8 [13.3-15.8]  (N=20) | 15.8 [14.1-16.3]  (N=18) | 0.271 | 0.33 [0.27-3.40]  (N=4) | 8.66 [2.30-8.73]  (N=9) | 0.185 |
| ROSC* | 14.2 [11.1-15.9]  (N=4) | 14.0 [12.1-15.7]  (N=11) | 0.949 | 0.27  (N=1) | 8.69 [3.89-9.71]  (N=6) | - |
| Survival at hospital admission* | 15.9 [13.3-16.1]  (N=4) | 13.9 [11.7-15.0]  (N=11) | 0.489 | 0.33 [0.30-0.36]  (N=2) | 8.73 [8.66-10.0]  (N=5) | 0.095 |
| Survival at hospital discharge* | 14.9 [11.1-16.1]  (N=8) | 13.9 [12.1-15.0]  (N=7) | 0.779 | 2.30 [0.40-8.66]  (N=5) | 9.38 [9.06-9.71]  (N=2) | 0.381 |

**Goal justification.**

There is no clear goal defined by standards or learned societies on clinical performance related to defibrillation efficacy [21]-[22]. In literature, monophasic shocks defibrillation efficacy is mainly superior to 70% whereas biphasic is superior to 80% [23]-[30]. Resuscitation guidelines define efficient defibrillation as cessation of shockable rhythm at 5s post-shock and proceed to state that beyond 5s, shock outcomes are biased by known confounders (call to AED connection time, bystander, cardiopulmonary resuscitation, drugs, initial VF…). Thus, it is consistent that AED performance should be assessed in terms of defibrillation efficacy right after the shock rather than in terms of return of spontaneous circulation or survival to hospital discharge. For all these reasons, we defined 80% as an acceptable goal read at 5s, for validation of defibrillation efficacy for patients in initial VF.
